# A collaborative translational research framework for evaluating and implementing the appropriate use of human genome sequencing to improve health

**DOI:** 10.1371/journal.pmed.1002631

**Published:** 2018-08-02

**Authors:** Muin J. Khoury, W. Gregory Feero, David A. Chambers, Lawrence E. Brody, Nazneen Aziz, Robert C. Green, A. Cecile J.W. Janssens, Michael F. Murray, Laura Lyman Rodriguez, Joni L. Rutter, Sheri D. Schully, Deborah M. Winn, George A. Mensah

**Affiliations:** 1 Office of Public Health Genomics, Centers for Disease Control and Prevention, Atlanta, Georgia, United States of America; 2 Maine-Dartmouth Family Medicine Residency Program, Augusta, Maine, United States of America; 3 Division of Cancer Control and Population Sciences, National Cancer Institute, NIH, Rockville, Maryland, United States of America; 4 National Human Genome Research Institute, National Institutes of Health, Bethesda, Maryland, United States of America; 5 Kaiser Permanente, Oakland, California, United States of America; 6 Brigham and Women’s Hospital, Broad Institute and Harvard Medical School, Boston, Massachusetts, United States of America; 7 Rollins School of Public Health, Emory University, Atlanta, Georgia, United States of America; 8 Yale School of Medicine, New Haven, Connecticut, United States of America; 9 *All of Us* Research Program, National Institutes of Health, Bethesda, Maryland, United States of America; 10 Office of Disease Prevention, National Institutes of Health, Bethesda, Maryland, United States of America; 11 Center for Translation Research and Implementation Science, National Heart, Lung, and Blood Institute, NIH, Bethesda, Maryland, United States of America

## Abstract

In a Policy Forum, Muin Khoury and colleagues discuss research on the clinical application of genome sequencing data.

**Summary points**

- There is currently insufficient scientific evidence to support routine nondiagnostic use of germline genome sequencing in healthcare settings and population screening, but an increasing number of health systems are piloting genomic sequencing projects for clinical care.
- In principle, numerous diagnostic or prognostic tests based on genes or variants could be used for different purposes across the life span, and an evidence-based approach is urgently needed to evaluate their possible clinical utility and facilitate appropriate implementation.
- We discuss a translational research framework that features collaboration among multiple health systems with already available genome sequencing data, intervention information, and clinical outcomes. The framework is based on evaluating the impact of genetic information on improving health outcomes with study designs that depend on the evolving level of evidence for specific intended clinical uses.
- In addition to observational studies, randomized controlled trials will be needed to assess health benefits, harms, and costs based on returning or not returning the results of selected genes/variants to patients, providers, or both, for specific clinical scenarios.
- The proposed approach will allow learning health systems to collect clinical utility evidence in a research environment and develop the necessary capacity for appropriate integration of sequencing alongside other medical services.

## Introduction

A vision for genomic medicine is that germline genome sequencing will be routinely conducted in health systems to provide healthcare and preventive services tailored to each individual [1]. For the most part, sequencing is not yet routinely used in clinical practice but is prioritized among people with certain diseases (e.g., ill newborns, and people with cancer or rare diseases) [2] or genetic predisposition to certain diseases (e.g., BReast CAncer susceptibility gene 1 [BRCA1] and BReast CAncer susceptibility gene 2 [BRCA2] testing for hereditary breast and ovarian cancer susceptibility) [3]. Recent studies have begun evaluating the use of genome sequencing for a wide variety of interventions in multiple healthcare settings and population groups. The idea that one test can provide a broad range of information on a vast number of conditions is unprecedented. Sequencing data could be used for patients’ immediate healthcare needs, but also for their future risk assessment and prevention for a wide variety of health issues and pharmaceutical management.

### A conundrum in evaluating the utility of genome sequencing for population health

Currently, there is limited direct evidence of clinical utility of germline genome sequencing to guide health service delivery and disease prevention in the population [4]. While we acknowledge the utility of genome sequencing in diagnosis and management of rare diseases, several ethical, legal, social, and economic concerns affect the use of sequencing in the general population [5]. Potential risks may include disappointment in information derived from the genome, regret about receipt of undesired information, loss of privacy, false expectations, misinterpretation, false reassurance, and potential for overscreening and unnecessary treatment [5].

Yet, several health systems in the United States and other countries [6] are beginning to integrate sequencing into patient care and disease prevention independent of disease, leading to unclear benefits, harms, and healthcare costs. In addition, direct-to-consumer genetic testing has been on the rise even with no or limited evidence of clinical validity and utility. Normally, evidentiary frameworks for genetic testing require establishing the clinical validity and utility of testing for a specific intended use [7]. This approach may present an insurmountable challenge in evaluating genome sequencing, as the human genome sequence can be used to answer numerous questions relevant to healthcare over time. Multiple “tests” can be used to direct healthcare-related activities (e.g., diagnosis, risk assessment, treatment, and prevention) for multiple diseases (e.g., heart disease, cancer) and can be deployed throughout life.

To address this challenge, some have advocated to focus sequencing efforts only on selected genes with evidence of clinical utility to guide practice [8]. Many genes, however, have established clinical associations but lesser evidence on clinical utility to guide healthcare decisions (e.g., many pharmacogenomic traits). Indeed, most human genes and associated variants participate in gene–gene and gene–environment interactions underlying common diseases, for which there is little evidence to support their use in routine clinical practice.

### How can we learn from the experience of genome sequencing among early adopters?

The early adoption of germline genome sequencing in practice may be viewed as an uncontrolled experiment, which could lead to both benefits and harms. However, it also provides a unique opportunity to develop an evidence-based process to accelerate evaluation and appropriate implementation. A fundamental question is whether or not we can use a genome sequencing platform embedded into learning health systems that can accelerate simultaneous evaluation of multiple testing scenarios with differing levels of evidence.

The topic of integrating genome sequencing in large health systems was discussed at a National Academy of Medicine workshop [9] that featured ongoing research initiatives in early adopter health systems in the US and around the world. Participants discussed evidentiary, economic, data sharing, and infrastructure requirements as well as outcome end points to measure success of implementation. The workshop highlighted ongoing research studies and systematic reviews that have attempted to evaluate the clinical actionability of genes and variants [10], and to study the implementation of sequencing in practice [11].

However, most work in this area is fragmented, based on small sample sizes, conducted in one health system at a time, or completely outside the healthcare systems by commercial and/or direct-to-consumer entities. The 100,000 Genomes Project in the United Kingdom [12] and the *All of Us* Research Program [13] (1 million participants) will contribute valuable knowledge in this space. Many of the elements discussed here will serve to inform such large studies as they return results of genome sequencing to participants.

We propose an evidence-based approach to accelerate the evaluation of clinical utility of genome sequencing and appropriate implementation in health systems. We posit that a large-scale translational research agenda with interrelated components can be built onto well-characterized populations with already available sequence data (in a biobank/research environment), risk factor information, intervention information, and clinical outcomes. The proposed framework calls for collaboration among several organizations, to recruit adequate numbers of individuals. This is crucial in order to understand the consequences of knowledge of multiple risk variants for multiple clinical scenarios. In addition to observational studies, randomized controlled trials (RCTs) can be designed to assess individual, family, system, and population outcomes based on returning versus not returning the results of selected genes/variants for specific clinical scenarios, depending on the existing level of evidence. This implies that the return of the results of genome sequencing to patients and providers will occur in a research controlled fashion, specified based on pre-agreed study protocols. The proposed framework will allow learning health systems to evolve the necessary capacity for appropriate integration of genomics alongside other medical services, such as screening and treatment, as well as to prepare the healthcare workforce and the public.

### A framework for accelerated evaluation and implementation of germline genome sequencing

The framework has five components, with a three-prong research agenda (Table 1) and two essential supporting activities. To fully vet this framework, additional input will be sought from multiple stakeholders to examine available approaches and develop specific recommendations for a collaborative research agenda.

**Table 1:** A translational multidisciplinary research framework to evaluate the clinical utility and implementation of genome sequencing by level of existing evidence.

Level of Evidence (Genes/Variants)	Examples	Research Framework	Research Questions
Tier 1*	HBOC, Lynch Syndrome, FH	Accelerated implementation science	Assessing patient, provider, and health systems success factors of optimal implementation and outcomes of existing recommendations, and reducing health disparities
Tier 2*	Selected pharmacogenetic traits, monogenic risk variants	Accelerated collaborative evaluation of clinical utility (RCTs)	Assessing benefits, harms, and costs from return of genomic information compared to standard of care; selected hybrid effectiveness/implementation studies to assess how genes and variants may be integrated into practice.
Tier 3*	Genetic risk scores, gene–environment interaction	Accelerated collaborative evaluation of clinical validity and utility	Assessing added value of using genomic information compared to existing approaches (prediction, discrimination, interventions, outcomes)**

*Tier 1: evidence of clinical validity and utility; tier 2: evidence of clinical validity but unclear utility; tier 3: unclear validity and utility (based on the paper by Dotson and colleagues [15]).

**Tiers 2 and 3 genes/variants also provide an opportunity to evaluate how to prepare a health system to anticipate new findings, both positive and negative, related to genome sequencing—what ancillary studies can focus on current use of genome sequencing, factors affecting uptake/de-implementation as warranted, so that future discoveries can be integrated into the learning health system.

Abbreviations: FH, familial hypercholesterolemia; HBOC, hereditary breast and ovarian cancer; RCT, randomized controlled trial.

1 Ongoing knowledge integration of the human genome

An ongoing knowledge integration process will be needed to drive genomic medicine translation research. As proposed by Berg and colleagues [14], knowledge integration entails developing, using, and updating ongoing systematic evaluations to “bin” the genes and genetic variants according to level of evidence. Interventions identified by such a process of having high potential for health benefit can then be prioritized for future study using approaches appropriate to the levels of existing evidence.

An example of an ongoing effort in evaluating clinical validity is the Clinical Genome Resource (ClinGen) [9]. ClinGen builds a central resource for evaluating clinical relevance of genes and variants [8]. Key goals are to develop and document an evidence-based process for curating the clinical interpretation of genomic variant associations, to assess clinical actionability and to disseminate collective knowledge and resources. Another example of knowledge integration incorporating assessment of clinical utility is based on a simple three-tier classification system proposed by Centers for Disease Control and Prevention (CDC) to help prioritize the genome and its applications for further translational research [15].

- Tier 1 genes and variants that have synthesized evidence with an evidence-based guideline that supports implementation in clinical practice. These genes are ready for an accelerated implementation science agenda (see below).
- Tier 2 genes and variants that have strong clinical validity information (i.e., validated gene–disease associations) but limited or no evidence for clinical utility (i.e., improved health outcomes if genomic information is used in practice) to support implementation in practice. These genes/variants should be targeted for accelerated test/drug development and research evaluation of clinical utility. In addition, hybrid effectiveness/implementation studies could be designed to assess how these genes and variants may be incorporated into recommendations for clinical practice.
- Tier 3 genes and variants include the majority of genes and variants with insufficient evidence on clinical validity or utility. These genes and variants can be targeted for accelerated evaluation of both clinical validity and utility. Tier 3 also contains variants that have guidelines against their use. These would not be addressed further and could, as evaluations mature, allow ineffective technologies to be removed from use (de-implemented).

2 Accelerated implementation science agenda for tier 1 genes/variants

Implementation of evidence-based tier 1 genes/variants will require enhanced services associated with the management of sequencing results (e.g., laboratory practice quality assurance and the availability of tailored interventions). Implementation science is a well-established field that is increasingly applied to genomic medicine research [16], which evaluates factors at multiple levels (patients, providers, health systems, electronic health records, state and federal policies) that can promote or impede the integration of evidence-based interventions into routine clinical and public health practice. A fundamental tenet of implementation science is that research and practice can coexist and indeed should be integrated to improve health outcomes. Research efforts within healthcare systems can inform the full spectrum of research needs, from discovery to implementation and back [17]. An important component of implementation science is to evaluate and address disparities in implementation and the consequences these disparities have at the population level [18].

Implementation science is typically used when high levels of evidence of benefit from the use of a new technology exist. As an example, we discuss the 10 genes relevant to three autosomal dominant high-penetrance conditions that seem immediately appropriate for implementation science research: hereditary breast and ovarian cancer risk associated with BRCA 1 and BRCA2 mutations, Lynch syndrome (colorectal, endometrial, and other cancers) associated with mutations in mismatch repair genes, and familial hypercholesterolemia (FH) associated with dysfunctional cholesterol processing. Each of these inherited conditions has evidence-based recommendations that are currently underutilized in most healthcare settings. The adoption of these recommendations will reduce the burden of heart disease or cancer [19]. In aggregate, these conditions are carried by more than 2 million people in the US, most of whom do not know that they are at markedly elevated risk for cancer or heart disease. Once patients are identified, cascade screening to relatives can be potentially valuable. However, it is likely to require consent from affected individuals and be limited by the number of relatives who can be contacted and agree to participate. Current recommendations for genetic testing exclude the general population and focus on subsets of the population based on family history, ethnicity, or other characteristics. Genome sequencing will allow the simultaneous study of implementation of existing recommendations while learning, in a research environment, new information on disease penetrance and interventions in individuals not covered by the existing guidelines.

For example, current USPSTF BRCA guidelines recommend counseling and testing based on high-risk family history and ethnicity [3]. Studying the return of genomic results in the absence of family history in large primary care settings will allow us not only to assess how to implement existing guidelines but also to learn new information on penetrance, actionability, and interpretation of variants of unknown significance in *BRCA1*, *BRCA2*, and other genes.

For FH, cascade screening can prevent early heart disease in affected relatives [20]. Cascade screening using phenotypic and genetic testing has been recommended by multiple groups for the purpose of early detection and treatment of affected relatives. However, optimal interventions for the implementation of cascade screening for FH have not been well studied [20].

3 Accelerated clinical utility research for tier 2 genes and variants

A collaborative translational research agenda can also accelerate the evaluation of and implementation studies for genetic variants that have robust and validated associations and have the potential for clinical utility. This category includes many monogenic risk variants as well as selected pharmacogenetic traits that are prevalent in the population, with potential relevance to clinical management of several commonly used drugs (e.g., clopidogrel, warfarin, statins, antidepressants). Collaborative groups such as the Clinical Pharmacogenetics Implementation Consortium (CPIC) and the Pharmacogenomics Knowledgebase (PharmGKB) evaluate emerging evidence of pharmacogenomics and publish recommendations to inform clinical practice [21]. Such efforts provide online resources that facilitate the interpretation of genetic test results and provide prescribing recommendations for specific gene–drug pairs. PharmGKB contains a list of many examples of tier 2 variants and provides information about how these genetic variations affect response to medications. As of May 25, 2018, PharmGKB has information on 65 “very important pharmacogenes,” 100 dosing guidelines, on 641 drugs, related to 130 drug pathways [21]. Given the frequency of use of drugs in healthcare, most people in the population have one or more pharmacogenomic variants related to guidelines that could become relevant to their care over a lifetime. There are a few well-defined pharmacogenomic traits for which testing has been recommended (e.g., Human Leukocyte Antigen [HLA]-B57 and Abacavir use in HIV treatment), and many more are tier 2 applications.

Additionally, when healthy people undergo genome sequencing, results of pharmacogenomic tier 2 genes/variants will be available to be evaluated for clinical utility if patients need certain medications. The value of preemptive pharmacogenomic testing has been suggested but not validated [22]. Many pharmacogenomic variants are sufficiently prevalent in the population to allow for a collaborative evaluation of their clinical utility for common drug exposures. For example, the evidence supporting the use of pharmacogenomics testing for warfarin has been limited [23]. Using the proposed framework, collaborative RCTs of return of pharmacogenomic results to patients and physicians, versus no return, could provide an accelerated evaluation of the potential benefits, harms, and costs of these tests in the context of specific clinical scenarios.

4 Accelerated evaluation of validity and utility of tier 3 genes and variants

Most human genes and their variants currently reside in a tier 3 bin, for which additional research will be needed to establish both clinical validity and utility. Collaborative multi-institution genome-wide association studies (GWAS) have provided a firm foundation for establishing gene–disease associations for numerous diseases [24]. So far, most variants associated with traits in GWAS have limited positive and negative predictive value and may have limited clinical utility. With the advent of genome sequencing, along with rapidly progressing efforts in measuring other “omic” analytes (e.g., DNA methylation, RNA expression, metabolomics), the need for accelerated research evaluation of predictive models using many biomarkers is more important than ever. For example, the Trans Omics Precision Medicine (TOPMed) research program [25] generates scientific resources and evidence to accelerate advances in precision medicine. The program collects whole-genome sequence and other -omics data in large cohorts. In addition, the Clinical Sequencing Exploratory Research (CSER) Consortium has produced pilot data on sequencing of healthy individuals as well as sequencing for various indications with return of secondary findings [26].

A collaborative effort across multiple institutions that are already generating data on low effect size variations in large numbers of individuals will offer immediate opportunities to accelerate joint analyses to evaluate the validity and utility for selected tier 3 genes/variants included in genetic risk scores. One example is the use of genetic risk scores in practice [27]. Genetic risk scores for various common diseases have been developed but have largely undefined clinical validity and utility in unselected populations. Collectively, analyses of genetic risk scores have shown that the contribution of multiple variants will be limited in predicting disease due to small effects of individual variants on disease risk [28]. Nearly all individuals are at slightly increased or slightly decreased genetic risk for any given disease, as compared with the average population risk. However, for people at the extremes of the distribution, disease risk can be markedly increased or decreased.

For example, in the context of heart disease, Knowles and Ashley recently discussed the emerging promise of genetic risk scores for a field in which the use of risk factors for clinical decision-making has had a long history in medicine [29]. They discuss how new studies based on hundreds of thousands of people and millions of genetic variants indicate that genetic risk scores may be able to add value to traditional risk factors in risk prediction. For example, Khera and colleagues [30] have shown that genetic risk scores can identify 2.5% of all individuals with a 4-fold increased risk for coronary disease that is similar to monogenic disease risk. They also observed similar patterns with genetic risk scores for breast cancer and severe obesity [30]. The implications of these findings for clinical practice and disease interventions will require additional studies. For example, can people with high scores be identified through other means (e.g., simple cholesterol tests, environmental risk factors, or simple family history)? Would such individuals require interventions (i.e., statin treatment)? Furthermore, genetic risk-stratification models must have adequate discrimination and calibration, and should produce several strata of the population for which different management strategies are needed and available to improve population health outcomes. A major translational challenge is to assess the added value of such testing compared with or in addition to existing disease screening or intervention approaches based on age, family history, and interacting environmental risk factors. Randomized trials could also be done to assess the potential benefits and harms of returning versus not returning results for genetic risk scores to providers and patients to accelerate evaluation of their use in practice.

5 Enhanced development of workforce, tools, and resources

An accelerated translational research agenda for evaluating genome sequencing in healthcare and population screening will require several “drivers” [31]. For this effort to succeed, a collaborative effort is crucial for data sharing and development of tools and resources for researchers, practitioners, policy makers, and the public. Ongoing efforts will be needed to enhance provider competency [32,33] and develop decision support tools and structured sequencing test results connected with electronic health records [34]. A common knowledge integration process will drive implementation studies for tier 1 genes/variants, evaluation studies for tier 2 genes/variants, and collaborative analyses and hypothesis generation/testing for tier 3 genes/variants. A common protocol with centralized review will be needed. Key features of resource development will be to engage various stakeholders, such as providers, payers, patients, and researchers [35]. Opportunities for workforce training in implementation science will be needed to enhance education for researchers and practitioners. Training opportunities in implementation science are available, including in-person and online courses, short workshops, and full academic programs.

## Discussion

We have described essential elements of a collaborative translational research agenda to evaluate the potential use of germline genome sequencing in primary care and population screening. There is some urgency to developing this agenda, as genome sequencing is becoming more reliable, less expensive, and widely used inside and outside healthcare systems, without a thorough investigation of its clinical utility.

The emphasis on binning genes/variants is based on independent evaluation of evidence for clinical utility and may not readily take into account multiple domains of genetic information over a lifetime of healthcare decisions. Also, aggregating genome data across populations and over time creates a challenge for evidence acquisition that requires an ongoing collaboration. In addition, there will be a need for additional research studies for other domains of genomic information, such as studying healthy newborns and children, and for carrier testing and reproductive purposes. Ongoing studies such as the MedSeq project [36] are providing important information on RCT designs that measure outcomes in multiple domains. Recent analyses from MedSeq [37] showed that adding genome sequencing to primary care can reveal new findings that have potential clinical utility. Providers were able to manage sequencing results appropriately. Additional MedSeq data revealed that sequencing did not significantly affect healthcare costs within the first 6 months of follow-up [36]. Nonetheless, RCT studies in primary care also illustrate design challenges, including the number of study arms required, difficulties in patient recruitment and retention, and measurement of multiple outcomes over a long period of time.

The proposed approach is focused on healthcare and population screening settings that require integration of genome sequencing with health services. As such, it requires a high evidence bar. Clearly, there is growing interest in genetics by the public, as exemplified by the growth of direct-to-consumer genetic testing. The popular interest in genomic information should be factored into the evaluation of impact and outcomes of various studies suggested as part of this framework.

A major challenge in implementing a research framework such as this is to motivate participation from various stakeholders. Also, clear lines of demarcation will have to be drawn between the “research” and “clinical practice” arms of such a collaboration [38,39]. For example, there has to be agreement on what genes/variants to return to participants and their providers and medical records, in the course of routine clinical practice. As applications from the human genome sequence move to tier 1, they could be integrated into a practice environment where evidence generation continues to occur. For tier 2 and 3 genes, the return of results will have to be done in the appropriate research environment (e.g., an RCT on the use of pharmacogenomics variants for certain medications). As evidence accumulates over time, an important question is how do health systems handle changing information and continuously reevaluate new information? Once integrated into care, it is notoriously difficult to de-implement practices that are subsequently found to lack effectiveness. In addition, ethical, legal, and social implications and other challenges lie ahead, necessitating community engagement to ensure that underrepresented groups are not left behind and to sustain the collection of data to inform the development of appropriate regulations and standards for test utilization, patient privacy, and data security.

## Conclusions

A robust large-scale translational collaborative effort is now needed to understand the health benefits and potential harms and costs of genome sequencing, by studying the implementation of what we know can work, evaluating the possible utility of promising genes and variants, and critically assessing the validity of emerging genomic information for improving health and preventing disease. Although details on how to accomplish this task are beyond the scope of this piece, we plan to assemble a transdisciplinary, multi-sectoral group of experts to identify compelling questions, examine the available data, explore critical challenges and opportunities, and develop specific recommendations to implement a collaborative research framework for accelerating the evaluation of human genome sequencing in clinical practice.

## Abbreviations

BRCA1: BReast CAncer susceptibility gene 1
BRCA2: BReast CAncer susceptibility gene 2
CDC: Centers for Disease Control and Prevention
ClinGen: Clinical Genome Resource
CPIC: Clinical Pharmacogenetics Implementation Consortium
CSER: Clinical Sequencing Exploratory Research
FH: familial hypercholesterolemia
GWAS: genome-wide association study
HLA: Human Leukocyte Antigen
PharmGKB: Pharmacogenomics Knowledgebase
RCT: randomized controlled trial
TOPMed: Trans-Omics for Precision Medicine
USPSTF: United States Preventive Services Task Force
