# Correction: A collaborative translational research framework for evaluating and implementing the appropriate use of human genome sequencing to improve health

**DOI:** 10.1371/journal.pmed.1002650

**Published:** 2018-08-16

**Authors:** Muin J. Khoury, W. Gregory Feero, David A. Chambers, Lawrence C. Brody, Nazneen Aziz, Robert C. Green, A. Cecile J. W. Janssens, Michael F. Murray, Laura Lyman Rodriguez, Joni L. Rutter, Sheri D. Schully, Deborah M. Winn, George A. Mensah

The fourth author’s name is incorrect. The correct name is Lawrence C. Brody. The correct citation is: Khoury MJ, Feero WG, Chambers DA, Brody LC, Aziz N, Green RC, et al. (2018) A collaborative translational research framework for evaluating and implementing the appropriate use of human genome sequencing to improve health. PLoS Med 15(8): e1002631. https://doi.org/10.1371/journal.pmed.1002631.
